# Episodic Therapy for Genital Herpes in Sub-Saharan Africa: A Pooled Analysis from Three Randomized Controlled Trials

**DOI:** 10.1371/journal.pone.0022601

**Published:** 2011-07-25

**Authors:** Helen A. Weiss, Gabriela Paz Bailey, Sam Phiri, Gerard Gresenguet, Jerome LeGoff, Jacques Pepin, David A. Lewis, Laurent Belec, Irving F. Hoffman, William C. Miller, Philippe Mayaud

**Affiliations:** 1 London School of Hygiene and Tropical Medicine, London, United Kingdom; 2 Del Valle University of Guatemala, Guatemala and Tephinet Inc, Decatur, Guatemala; 3 Lighthouse Trust, Lilongwe, Malawi; 4 Centre National de Référence pour les Maladies Sexuellement Transmissibles, Bangui, Central African Republic; 5 Paris Diderot University and Laboratoire de Virologie, Hôpital Saint-Louis, Assistance Publique Hôpitaux de Paris, Paris, France; 6 Université de Sherbrooke, Sherbrooke, Canada; 7 National Institute for Communicable Diseases, National Health Laboratory Service, Johannesburg, South Africa; 8 University of the Witwatersrand, Johannesburg, South Africa; 9 University of North Carolina, Chapel Hill, North Carolina, United States of America; University of Toronto, Canada

## Abstract

**Background:**

A randomized controlled trial in South Africa found a beneficial effect of acyclovir on genital ulcer healing, but no effect was seen in trials in Ghana, Central African Republic and Malawi. The aim of this paper is to assess whether the variation in impact of acyclovir on ulcer healing in these trials can be explained by differences in the characteristics of the study populations.

**Methodology/Principal Findings:**

Pooled data were analysed to estimate the impact of acyclovir on the proportion of ulcers healed seven days after randomisation by HIV/CD4 status, ulcer aetiology, size and duration before presentation; and impact on lesional HIV-1. Risk ratios (RR) were estimated using Poisson regression with robust standard errors. Of 1478 patients with genital ulcer, most (63%) had herpetic ulcers (16% first episode HSV-2 ulcers), and a further 3% chancroid, 2% syphilis, 0.7% lymphogranuloma venereum and 31% undetermined aetiology. Over half (58%) of patients were HIV-1 seropositive. The median duration of symptoms before presentation was 6 days. Patients on acyclovir were more likely to have a healed ulcer on day 7 (63% vs 57%, RR = 1.08, 95% CI 0.98–1.18), shorter time to healing (p = 0.04) and less lesional HIV-1 RNA (p = 0.03). Small ulcers (<50 mm^2^), HSV-2 ulcers, first episode HSV-2 ulcers, and ulcers in HIV-1 seropositive individuals responded best but the better effectiveness in South Africa was not explained by differences in these factors.

**Conclusions/Significance:**

There may be slight benefit in adding acyclovir to syndromic management in settings where most ulcers are genital herpes. The stronger effect among HIV-1 infected individuals suggests that acyclovir may be beneficial for GUD/HIV-1 co-infected patients. The high prevalence in this population highlights that genital ulceration in patients with unknown HIV status provides a potential entry point for provider-initiated HIV testing.

## Introduction

Herpes simplex virus-2 (HSV-2) is the most common cause of genital ulcer disease (GUD) worldwide [1]. The natural course of genital herpes consists of subclinical genital viral shedding and clinical recurrences of blisters and ulcers which occur on the vulva, vagina or cervix in women, and on the glans, prepuce or shaft of the penis in men. The first episode of genital herpes is usually the most severe, lasting 2 to 4 weeks if untreated, and subsequent recurrences last 7 to 12 days without treatment [2]. A study conducted in the 1980s reported that oral acyclovir significantly reduced viral shedding, improved ulcer healing and decreased the incidence of new lesion formation among patients with recurrent genital herpes who received treatment within 48 hours of the onset of lesions [3]. A similar effect was seen on first episode genital herpes [4]. In 2003, the World Health Organization (WHO) published sexually transmitted infections (STI) treatment guidelines recommending addition of acyclovir to syndromic management of GUD in countries where the prevalence of HSV-2 as a GUD aetiology exceeds 30% [5]. However, a recent survey conducted in eight African countries found that acyclovir was rarely available in public health facilities, mainly due to cost and regulatory barriers [6].

The effectiveness of adding acyclovir to syndromic management for GUD in Africa was recently evaluated in three randomized controlled trials (RCTs). Two of the trials, one among women in Ghana and Central African Republic (CAR) and one among both men and women in Malawi, found no impact of standard dose acyclovir on ulcer healing [7]–[8]. However, a trial among South African men found that healing was significantly faster among those in the acyclovir arm (61% vs 42% healed at day 7; p = 0.003) [9] and acyclovir is now included in the guidelines for first-line management of GUD in South Africa [10].

In this paper, we analyze pooled data from the three RCTs to assess whether the variation in impact of acyclovir on ulcer healing can be explained by differences in the characteristics of the study populations in this large series of patients presenting with GUD in four distinct African settings.

## Methods

Details of the three individual trials have been reported elsewhere [7]–[9] (Trials registration numbers: NCT00158483, ISRCTN32121857, NCT00164424). The trials had similar designs, with an *a-priori* plan to conduct pooled analyses. Briefly, in each trial, consenting consecutive patients with clinically confirmed GUD were enrolled and randomized to either acyclovir or placebo (400 mg three times daily for 5 days for Ghana/CAR and South Africa; 800 mg two times daily for 5 days in Malawi). Patients with very large ulcers (surface area ≥500 mm^2^) and those with long ulcer duration (>21 days) were not included in the trial in Ghana/CAR and were treated immediately with acyclovir instead. Participants were asked to return for follow-up on day 2 and/or 4, day 7, day 10 (South Africa only), day 14 and day 28.

The primary outcomes in this analysis were the impact of acyclovir on the proportion of ulcers healed and lesional HIV-1 RNA (in HIV-1 seropositive patients) at 7 days post-randomization (defined as visits occurring on days 6–8 inclusive). The secondary outcome was time to ulcer healing over the 28 days of follow-up.

Ulcer healing was defined as a reduction in the size (in mm^2^) of the largest ulcer at baseline by at least 90%. Further analyses used absolute ulcer size (<10 mm^2^) as the outcome. Thresholds for detectable lesional HIV-1 RNA were 50 copies/mL for South Africa, 250 copies/mL for Ghana/CAR, and 400 copies/mL for Malawi. Ulcers which were not sampled because they were healed were assumed to have undetectable lesional HIV-1 RNA.

Sub-groups of interest were defined *a-priori* as follows:

- Ulcer aetiology (HSV ulcer, non-HSV ulcer, first episode HSV-2 ulcer, recurrent HSV-2 ulcer). First episode HSV-2 ulcers are defined as HSV-2 seronegative patients with HSV-2 DNA detected in the lesion. Six patients who had an HSV-1 ulcer without evidence of lesional HSV-2 infection were included as HSV ulcers.
- Duration of symptoms before presentation (≤2 days, 3–4 days, 5–6 days, > = 7 days).
- Size of ulcer at baseline (<50 mm^2^, ≥50 mm^2^).
- HIV/CD4 status: (HIV seronegative, HIV-1 seropositive with CD4 >500 cells/mm^3^; HIV-1 seropositive with CD4 200–500 cells/mm^3^; HIV-1 seropositive with CD4 <200 cells/mm^3^). Too few patients were on antiretroviral therapy to analyse these separately.

Statistical analyses were conducted in Stata 11.0 (StataCorp, Texas, USA). Poisson regression with robust standard errors was used to estimate risk ratios (RR) and 95% confidence intervals (CI) [11]. The risk difference and 95%CI for the primary analysis was also estimated, using binomial methods. Time-to-ulcer-healing was assessed graphically using Kaplan-Meier analysis and a log-rank test was used to obtain a p-value for a difference in time-to-healing by arm. The Kruskal-Wallis test was used to compare median durations between study sites. To investigate whether the differential effect of acyclovir in South Africa could be explained by factors associated with ulcer healing, we stratified analyses by South Africa versus non South Africa sites and tested for effect-modification by site. To allow for imbalances between arms, risk ratios of the impact of acyclovir were adjusted for factors potentially associated with ulcer healing (ulcer aetiology, size, duration of symptoms before enrolment and HIV status).

### Ethics statement

Each trial was approved by the ethics committee of the London School of Hygiene and Tropical Medicine, and the following local ethics committees and institutional review boards: ANRS 1212 - ethics committees of the Ministries of Health of Ghana and the Central African Republic; Malawi trial - National Health Sciences Research Committee in Malawi and the ethics committees of University of North Carolina and the US Centers for Disease Control and Prevention (CDC); South Africa - ethics committees and institutional review boards of the University of the Witwatersrand, the CDC, and the Gauteng Provincial and City of Johannesburg Health Departments. All participants provided written informed consent.

## Results

### Characteristics of GUD patients

**Figure 1:**
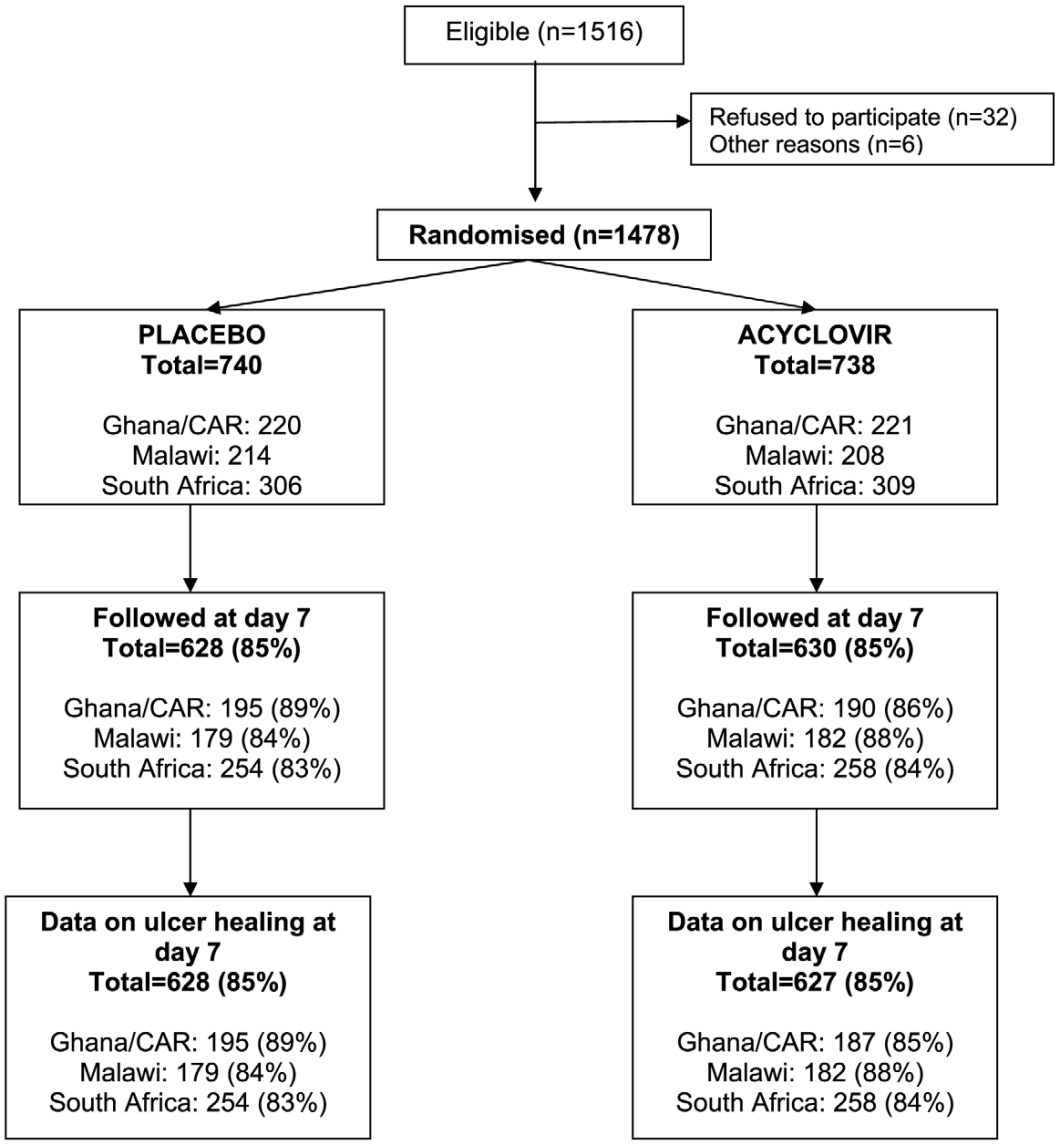
CONSORT flow diagram of eligibility, enrolment and follow-up in three African randomized controlled trials of episodic therapy for genital herpes.

Of the 1516 GUD patients eligible for the three trials, 1478 were randomized (550 female and 928 male). The flow of participants through the trials is shown in Figure 1. Sociodemographic and behavioural characteristics of the participants are shown in Table 1 (by trial arm) and Table 2 (by site). There was good balance in characteristics by trial arm (Table 1). The median age of participants was 28 years (interquartile range 24–34 years), and this was similar by study site and sex. Overall, almost half of men (47%) and one-third of women (33%) reported having had a previous GUD in the past year (Table 2). At the enrolment visit, the median number of ulcers per patient was two in each country, and women in Ghana and CAR tended to have fewer ulcers than patients in Malawi and South Africa. The size of the largest ulcer was measured for each patient, and varied considerably, with median size being smallest in Malawi (21 mm^2^ and 28 mm^2^ respectively in women and men), and largest in Ghana and CAR (147 mm^2^ and 130 mm^2^, respectively), despite exclusion of ulcers ≥500 mm^2^ in these sites. The median time from first symptoms to presentation was 6 days, and this was slightly shorter in South Africa and Ghana (5 days) than among women in CAR and women in Malawi (7 days; p = 0.0001). Overall, 28% of women and 13% of men were on antibiotics for the current ulcer, including 43% of women in CAR.

**Table 1:** Demographic, behavioural and clinic characteristics of 1478 trial participants with genital ulcer disease (GUD) at enrolment, by trial arm.

	Placebo arm	Acyclovir arm	Total
Total	N = 740	N = 736	N = 1478
	n (%)	n (%)	n (%)
**Male**	465 (63%)	463 (63%)	928 (63%)
**Age, years**			
15–24	191 (26%)	210 (29%)	401 (27%)
25–34	368 (50%)	368 (50%)	736 (50%)
> = 35	181 (25%)	158 (21%)	339 (23%)
**Marital status**			
Single	356 (48%)	344 (47%)	700 (47%)
Married	292 (39%)	305 (41%)	597 (40%)
Sep/div/widow	92 (12%)	87 (12%)	179 (12%)
**Education**			
Primary or lower	274 (37%)	289 (39%)	563 (38%)
Secondary or higher	464 (63%)	447 (61%)	911 (62%)
**Number of partners in last 3 months**			
None	154 (21%)	155 (21%)	309 (21%)
1	387 (53%)	383 (52%)	770 (52%)
2–9	165 (22%)	157 (23%)	332 (23%)
> = 10	30 (4%)	29 (4%)	59 (4%)
**New partner in last 3 months** a	131 (30%)	137 (32%)	268 (31%)
**Sex for money/gifts** b	150 (20%)	136 (18%)	286 (19%)
**GUD in past year**	313 (43%)	294 (40%)	607 (42%)
**Number of ulcers**			
1	263 (36%)	269 (37%)	532 (37%)
2	158 (22%)	164 (23%)	322 (22%)
3	122 (17%)	90 (12%)	212 (15%)
> = 4	184 (25%)	204 (28%)	388 (27%)
**Size of largest ulcer,mm^2^**			
<50	381 (52%)	404 (55%)	785 (53%)
50–149	189 (26%)	190 (26%)	379 (26%)
150–299	111 (15%)	89 (12%)	200 (14%)
300–500	55 (7%)	51 (7%)	106 (7%)
**Duration of symptoms at presentation**			
1–2 days	45 (6%)	51 (7%)	96 (7%)
3–4 days	204 (28%)	222 (31%)	426 (29%)
5–6 days	128 (18%)	110 (15%)	238 (16%)
1 week +	351 (48%)	340 (47%)	691 (47%)
**On antibiotics**	143 (19%)	132 (18%)	275 (19%)
**HSV ulcer**	471 (65%)	459 (63%)	930 (64%)
**HIV-1 seropositive**	433 (59%)	414 (56%)	847 (58%)

a This question was not asked in the South African trial.

b For Ghana and CAR, this is current occupation as a sex worker. In Malawi and South Africa, the question refers to ever having exchanged sex for money.

**Table 2:** Demographic, behavioural and clinic characteristics of 1478 trial participants with genital ulcer disease (GUD) at enrolment, by country and sex.

	Ghana Females	CAR Females	Malawi Females	Malawi Males	S Africa Males	All females	All males
Total	N = 284	N = 157	N = 109	N = 313	N = 615	N = 550	N = 928
	n (%)	n (%)	n (%)	n (%)	n (%)	n (%)	n (%)
**Age, years**							
15–24	110 (39%)	48 (31%)	40 (37%)	73 (23%)	130 (21%)	198 (36%)	203 (22%)
25–34	104 (37%)	75 (48%)	48 (44%)	186 (59%)	323 (53%)	227 (41%)	509 (55%)
> = 35	68 (24%)	34 (22%)	21 (19%)	54 (17%)	162 (26%)	123 (22%)	216 (23%)
**Median (IQR** a **)**	27 (23–34)	29 (23–34)	28 (23–33)	28 (25–33)	29 (25–35)	28 (23–34)	29 (25–34)
**Marital status**							
Single	163 (58%)	52 (33%)	16 (15%)	115 (37%)	354 (58%)	231 (42%)	469 (51%)
Married	72 (26%)	60 (38%)	62 (57%)	164 (52%)	239 (39%)	194 (35%)	403 (43%)
Sep/div/widow	47 (17%)	45 (29%)	31 (28%)	34 (11%)	22 (4%)	123 (22%)	56 (6%)
**Education**							
Primary or lower	131 (47%)	76 (48%)	59 (54%)	160 (51%)	137 (22%)	266 (49%)	297 (32%)
Secondary or higher	150 (53%)	81 (52%)	50 (46%)	153 (49%)	477 (78%)	281 (51%)	630 (68%)
**Number of partners in last 3 months**							
None	99 (35%)	130 (83%)	14 (13%)	20 (6%)	46 (8%)	243 (44%)	66 ((47%)
1	107 (38%)	22 (14%)	75 (69%)	185 (59%)	381 (62%)	204 (37%)	566 (61%)
2–9	22 (8%)	3 (2%)	19 (17%)	108 (35%)	180 (30%)	44 (8%)	288 (31%)
> = 10	54 (19%)	2 (1%)	0 (0%)	0 (0%)	3 (0.5%)	56 (11%)	3 (<1%)
**New partner in last 3 months** b	79 (28%)	10 (6%)	21 (19%)	158 (50%)	n/a	110 (20%)	158 (50%)
**Sex for money/gifts** c	59 (21%)	8 (5%)	21 (19%)	119 (38%)	79 (13%)	88 (16%)	198 (21%)
**Reported GUD in past year**	79 (28%)	63 (40%)	40 (37%)	117 (37%)	308 (52%)	186 (33%)	425 (47%)
**Number of ulcers**							
1	117 (42%)	70 (45%)	31 (28%)	100 (32%)	214 (36%)	218 (40%)	314 (35%)
2	79 (28%)	45 (29%)	26 (24%)	58 (19%)	114 (19%)	150 (28%)	172 (19%)
3	54 (19%)	21 (13%)	16 (15%)	44 (14%)	77 (13%)	91 (17%)	121 (13%)
> = 4	28 (10%)	21 (13%)	36 (33%)	111 (35%)	192 (33%)	85 (16%)	303 (33%)
**Median (IQR)**	2 (1–3)	2 (1–3)	2 (1–4)	2 (1–5)	2 (1–4)	2 (1–3)	2 (1–4)
**Size of largest ulcer,mm^2^**							
<50	101 (36%)	18 (11%)	72 (66%)	200 (64%)	394 (64%)	191 (35%)	594 (64%)
50–149	50 (18%)	80 (51%)	30 (28%)	76 (24%)	143 (23%)	160 (29%)	219 (24%)
150–299	69 (24%)	48 (31%)	6 (6%)	22 (7%)	55 (9%)	123 (22%)	77 (8%)
300–500	62 (22%)	11 (7%)	1 (1%)	11 (4%)	21 (3%)	74 (14%)	32 (3%)
**Median (IQR)**	147(30–290)	130(80–195)	21(12–50)	28(12–75)	35(15–81)	100(28–221)	32(15–79)
**Duration of symptoms at presentation**							
1–2 days	22 (8%)	3 (2%)	5 (5%)	17 (5%)	49 (8%)	30 (6%)	66 (7%)
3–6 days	145 (52%)	46 (30%)	43 (42%)	141 (45%)	289 (48%)	234 (44%)	430 (47%)
1 week +	84 (30%)	45 (29%)	24 (23%)	67 (21%)	158 (26%)	153 (28%)	225 (25%)
2 weeks +	20 (7%)	32 (21%)	15 (15%)	40 (13%)	79 (13%)	67 (12%)	119 (13%)
3 weeks +	8 (3%)	29 (19%)	16 (16%)	48 (15%)	26 (4%)	53 (10%)	74 (8%)
**Median days (IQR)**	5 (3–7)	7 (5–15)	7 (4–14)	6 (6–14)	5 (3–8)	7 (4–10)	6 (4–10)
**On antibiotics**	60 (21%)	68 (43%)	24 (22%)	68 (22%)	55 (9%)	152 (28%)	123 (13%)

a Interquartile range.

b This question was not asked in the South African trial.

c For Ghana and CAR, this is current occupation as a sexworker. In Malawi and South Africa, the question refers to ever having exchanged sex for money.

HIV-1 seroprevalence was high in all sites, ranging from 34% among women in Ghana to 77% among Malawian women (Table 3). Among HIV-1 infected persons, mean HIV-1 plasma viral load was 4.77 log_10_ copies/mL (95% CI 4.71–4.84) overall, but varied significantly between site (p<0.0001), being lowest in Ghana (4.26 log_10_ copies/mL), where the proportion of participants with CD4 count above 500 cells/mm^3^ was also highest (38%). Overall, almost half of HIV-1 seropositive women (47%), and a third of HIV-1 seropositive men (34%) presented with CD4 counts below 200 cells/mm^3^. Despite this, the proportion of HIV-1 seropositive participants on antiretroviral therapy was low (9% in women, 3% in men). Overall, about half of the HIV-1 seropositive participants had detectable lesional HIV-1 RNA at enrollment, but this varied substantially by site (from 31% among women in Malawi to 80% in CAR; p<0.0001).

**Table 3:** HIV/HSV-related characteristics of trial participants with genital ulcer disease (GUD) at enrolment, by country & sex.

	GhanaFemales	CARFemales	Malawi Females	Malawi Males	S AfricaMales	All females	All males
Total	N = 284	N = 157	N = 109	N = 313	N = 615	N = 550	N = 928
	n (%)	n (%)	n (%)	n (%)	n (%)	n (%)	n (%)
**HIV-1 seropositive**	95 (34%)	108 (69%)	84 (77%)	173 (55%)	387 (63%)	287 (53%)	560 (60%)
**Mean plasma viral load, log_10_ copies/mL** a ** (95%CI)**	4.26(4.01–4.51)	4.95(4.71–5.19)	4.74(4.55–4.93)	4.90(4.78–5.02)	4.80(4.72–4.88)	4.66(4.53–4.80)	4.83(4.77–4.90)
**CD4 count** a ^,^ b, **cells/mm^3^**							
>500	28 (38%)	10 (24%)	6 (8%)	23 (15%)	59 (15%)	44 (23%)	82 (15%)
200–500	19 (26%)	10 (24%)	27 (36%)	80 (51%)	196 (51%)	56 (29%)	276 (51%)
<200	26 (36%)	22 (52%)	42 (56%)	54 (34%)	130 (34%)	90 (47%)	184 (34%)
**Median CD4 (IQR)**	361(138–611)	193(70–479)	173(84–347)	263(151–409)	282(165–419)	223(104–467)	279(164–417)
**On antiretrovirals** a ^,^ c	7 (7%)	12 (11%)	7 (8%)	5 (3%)	9 (3%)	26 (9%)	14 (3%)
**Detectable lesional HIV-1 RNA^1,^** d	11/31(35%)	84/105(80%)	24/78(31%)	82/164(50%)	173/379(46%)	119/214(56%)	255/543(47%)
**HSV-2 seropositive**	198 (71%)	149 (95%)	85 (81%)	214 (69%)	434 (71%)	430 (79%)	648 (70%)
**HSV ulcer (HSV-1 or HSV-2)**	120 (45%)	92 (59%)	83 (76%)	184 (59%)	451 (74%)	295 (56%)	635 (69%)
**HSV-2 ulcer**	119 (45%)	92 (59%)	83 (76%)	184 (59%)	446 (73%)	294 (55%)	630 (58%)
**First episode HSV-2 ulcer** e	19 (7%)	5 (3%)	10 (9%)	57 (18%)	134 (22%)	34 (6%)	191 (21%)
**HIV-1 seropositive with HSV-2 ulcer**	52 (19%)	64 (41%)	67 (61%)	112 (36%)	293 (48%)	183 (34%)	405 (44%)
**Detectable lesional HIV-1 RNA in HIV-1 seropositive patients with HSV-2 ulcers** f	9 (47%)	53 (84%)	21 (33%)	48 (44%)	120 (42%)	83 (57%)	168 (43%)

a Among HIV seropositive individuals.

b Missing CD4 data for 22/95 HIV positive women in Ghana; 66/108 in CAR: 9/84 women in Malawi,16/173 men in Malawi, 2/387 men in South Africa.

c Missing ARV data for 1/95 women in Ghana and 54/387 men in South Africa.

d Uses threshold of: 250 copies/mL for Ghana & CAR; 400 copies/mL for Malawi; 50 copies/mL for S Africa. Lesional HIV-1 RNA only.

e Defined as HSV-2 seronegative and HSV-2 DNA detected in the ulcer. Data are missing for 27 participants (21 with missing ulcer aetiology, 6 with missing HSV-2 serostatus).

f Among 541 HIV-1 seropositive individuals with HSV-2 ulcers and data on lesional HIV-1 RNA.

**Figure 2:**
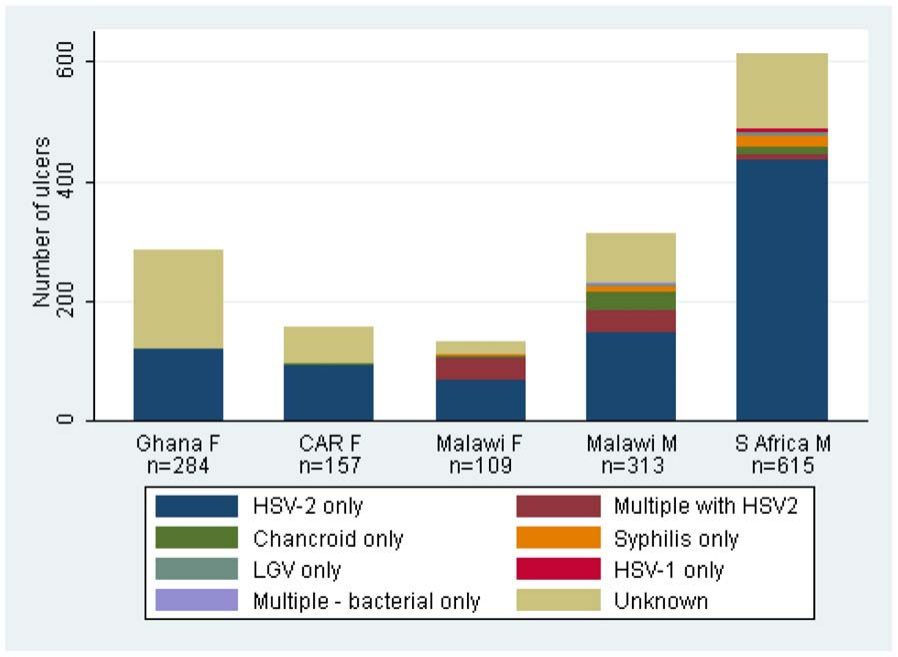
Ulcer aetiology in genital ulcer disease patients in Sub-Saharan African trials, by country and sex.

HSV-2 seroprevalence was 74% overall, and varied significantly by site, being highest among women in CAR (95%) and Malawi (81%) compared to women in Ghana and men in Malawi and South Africa (69–71%) (p<0.0001). Overall, about two-thirds of ulcers had detectable HSV DNA (type 1 or 2) alone or in combination with other pathogens, making this the most common ulcer aetiology (Figure 2). Of the 930 HSV ulcers, 6 had detectable HSV-1 DNA only (one from Ghana, 5 from South Africa), and the remaining 924 had detectable HSV-2 DNA. Of these, 225 (24%) ulcers were first episode HSV-2 ulcers, 693 (76%) had recurrent HSV-2 ulcers and the remaining 6 had unknown HSV-2 serostatus. A total of 154 participants (10.4%) had at least one lesional bacterial infection detected, and 90 participants (6.1%) had bacterial infections only. Prevalence of chancroid was 4.9% (n = 73), syphilis 3.5% (n = 52), and lymphogranuloma venereum 2.2% (n = 33), whilst 435 ulcers (30%) had undefined or unknown aetiology.

### Impact of acyclovir on ulcer healing

Of the 1478 patients enrolled, 1258 (85%) were seen on day 7, of whom 1255 had a genital examination (Figure 1). The proportion seen was similar by site (ranging from 82% among Malawian women to 89% in women in CAR) and by arm (85% in each arm).

**Figure 3:**
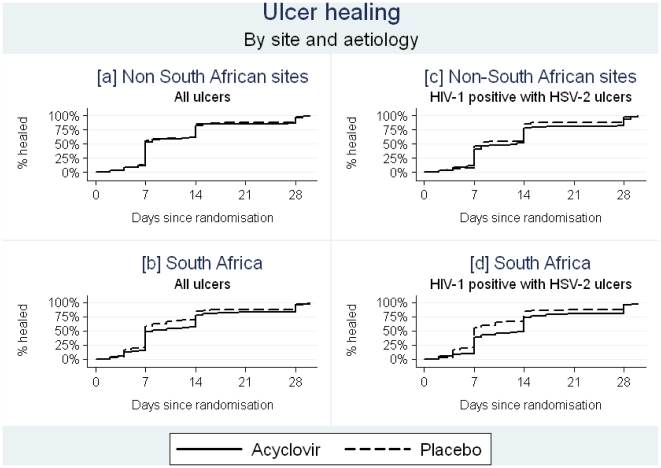
Time-to-ulcer-healing among all patients and those HIV-1 infected with HSV ulcers, by site.

Overall, there was a small impact of acyclovir on the proportion of ulcers healed on day 7 (63% vs. 57% in acyclovir and placebo arms respectively; RR = 1.10, 95% CI 1.00–1.20; risk difference = 5.7%, 95% CI 0.3%–11.1; Table 4). Adjustment for factors potentially associated with ulcer healing rate (GUD aetiology, size of ulcer at baseline, duration before presentation, and HIV/CD4 status) made little difference (adjusted RR (aRR) = 1.08, 95% CI 0.98–1.18). The impact on healing was greatest in South Africa (aRR = 1.21, 95% CI 1.05–1.40) compared with other sites (aRR = 0.98, 95% CI 0.87–1.10) with evidence of effect-measure modification of these adjusted risk ratios (p-interaction = 0.02). Overall, individuals randomized to the acyclovir arm had slightly faster healing (p = 0.04), principally due to the South African trial where time to healing was significantly improved (p = 0.007; Figure 3b). Acyclovir had little impact on time to healing in the other sites (p = 0.76; Figure 3a). Results were similar if ulcer size<10 mm^2^ was used as the outcome (results not shown).

**Table 4:** Impact of acyclovir on ulcer healing^1^ at Day 7 among 1255 trial participants.

	N (seen at Day 7)	Placebon (% healed)	Acyclovirn (% healed)	Risk ratio(95% CI)	Adjusted risk ratio (95% CI)^2^
Total		N = 628	N = 627		
**All patients**	**1255**	**358 (57%)**	**393 (63%)**	**1.10 (1.00–1.20)**	**1.08 (0.98–1.18)**
**Country and sex**				P-int^3^ = 0.02	P-int = 0.06
Ghana Females	243	99 (81%)	97 (80%)	0.99 (0.87–1.12)	1.00 (0.88–1.14)
CAR Females	139	25 (34%)	23 (35%)	1.02 (0.64–1.61)	0.80 (0.43–1.48)
Malawi Females	88	30 (70%)	32 (70%)	1.01 (0.76–1.31)	1.00 (0.73–1.35)
Malawi Males	273	72 (53%)	73 (54%)	1.02 (0.82–1.28)	0.99 (0.80–1.23)
South Africa Males	512	133 (52%)	168 (65%)	1.24 (1.07–1.44)	1.21 (1.05–1.40)
**By ulcer aetiology at presentation**				P-int = 0.04^4^	P-int = 0.04^4^
Non HSV ulcer	450	135 (62%)	141 (61%)	0.98 (0.84–1.13)	0.94 (0.81–1.09)
HSV ulcer	788	212 (53%)	246 (63%)	1.19 (1.06–1.34)	1.15 (1.03–1.29)
First episode HSV-2 ulcer	188	54 (56%)	61 (68%)	1.22 (0.98–1.54)	1.26 (1.00–1.57)
Recurrent HSV-2 ulcer	593	156 (52%)	183 (62%)	1.18 (1.03–1.36)	1.14 (1.00–1.30)
HSV-2 ulcer only	736	201 (53%)	231 (64%)	1.21 (1.07–1.36)	1.17 (1.04–1.32)
HSV-2 plus bacterial infection	46	11 (55%)	15 (58%)	1.05 (0.62–1.77)	0.94 (0.57–1.55)
**By duration before presentation**				P-int = 0.55	P-int = 0.35
1–2 days	78	26 (72%)	30 (71%)	0.99 (0.75–1.31)	1.09 (0.85–1.40)
3–4 days	360	115 (64%)	132 (73%)	1.15 (1.00–1.32)	1.11 (0.97–1.28)
5–6 days	209	69 (63%)	61 (62%)	0.98 (0.79–1.21)	0.93 (0.75–1.15)
≥7 days	587	141 (48%)	160 (54%)	1.13 (0.97–1.33)	1.14 (0.97–1.34)
**By size of ulcer at baseline**				P-int = 0.002	P-int = 0.002
<50 mm^2^	663	196 (60%)	253 (74%)	1.23 (1.11–1.37)	1.20 (1.08–1.34)
> = 50 mm^2^	590	162 (53%)	140 (49%)	0.91 (0.78–1.07)	0.88 (0.74–1.03)
**By HIV/CD4 status (cells/mm^3^)**				P-int = 0.13	P-int = 0.15
HIV uninfected	524	174 (69%)	188 (70%)	1.02 (0.91–1.14)	1.02 (0.90–1.14)
HIV-1 infected	726	182 (49%)	202 (57%)	1.16 (1.02–1.33)	1.16 (1.01–1.32)
HIV-1 infected, CD4≥500	101	33 (60%)	27 (59%)	0.98 (0.71–1.36)	0.99 (0.74–1.31)
HIV-1 infected, CD4 200–500	275	64 (49%)	91 (63%)	1.29 (1.04–1.60)	1.25 (1.02–1.53)
HIV-1 infected CD4<200	249	64 (50%)	62 (52%)	1.03 (0.81–1.32)	1.00 (0.79–1.27)

1 Defined as a > = 90% reduction in size of largest ulcer from enrolment.

2 Risk ratio for impact of acyclovir among people in that strata, adjusted for all other variables in the table (ulcer aetiology is categorised as non-HSV ulcer, HSV ulcer).

3 P-value for interaction.

4 Interaction between non-HSV and HSV ulcers.

As expected, acyclovir was associated with improved healing on day 7 among individuals with HSV ulcers compared to those with non-HSV ulcers (aRR = 1.15; 95% CI 1.03–1.29 and aRR = 0.94; 95% CI 0.81–1.09; p-interaction = 0.04; Table 4). However, the effect on healing of HSV ulcers was seen only in South Africa (South Africa: aRR = 1.30, 95% CI 1.09–1.55; other sites: aRR = 1.03, 95% CI 0.89–1.20; p-interaction = 0.05). The impact was strongest for first episode HSV-2 ulcers (aRR = 1.26, 95% CI 1.00–1.57), and again the effect was limited to South Africa (South Africa: aRR = 1.43, 95% CI 0.98–2.09; other sites: aRR = 1.07, 95% CI 0.83–1.37; p-interaction = 0.12).

There was little difference in impact of acyclovir by duration of symptoms before presentation, but acyclovir had a better impact on healing of small ulcers than larger ulcers (p-interaction = 0.002; Table 4). Again, differences persisted between South African and the other sites (aRR for small ulcers in South Africa = 1.33, 95% CI 1.13–1.55; aRR for small ulcers in other sites = 1.10, 95% CI 0.95–1.26; p-interaction = 0.09).

The effect of acyclovir was slightly stronger among HIV-1 seropositive individuals (aRR = 1.16, 95% CI 1.01–1.32; Table 4) than HIV-uninfected individuals (aRR = 1.02, 95% CI 0.90–1.14). The effect among HIV-1 seropositive individuals was largely due to an effect in South Africa (aRR = 1.31, 95% CI 1.06–1.60) rather than in other sites (aRR = 1.08, 95% CI 0.90–130), although this difference was not significant (p-interaction = 0.17). A similar pattern was seen when analyses were restricted to HIV-1 seropositive individuals with HSV-2 ulcers (Figure 3c and 3d). In this subgroup, there was evidence of an impact overall (aRR = 1.20, 95% CI 1.02–1.41) but this was only seen in South Africa (aRR = 1.46, 95% CI 1.16–1.85) and not in other sites (aRR = 0.98, 95% CI 0.78–1.2; p-interaction = 0.03).

### Impact of acyclovir on lesional HIV-1 RNA

Of the 726 HIV-1 seropositive individuals who were seen on Day 7, data on lesional HIV-1 RNA was available for 667 (92%) (Table 5). Overall, there was evidence of an impact of the intervention on detectable lesional HIV-1 RNA after adjusting for ulcer aetiology, size of ulcers, duration of symptoms and CD4 group (aRR = 0.73, 95%CI:0.55–0.98). Again, the impact was confined to South Africa (aRR = 0.58, 95% CI 0.39–0.84), with no evidence of an effect at other sites (aRR = 1.01, 95% CI 0.65–1.57; p–interaction = 0.05; results not shown).

**Table 5:** Impact of acyclovir on lesional HIV-1 RNA on Day 7 among 667 HIV-1 seropositive trial participants.

	N	Placebon (% lesional HIV)	Acyclovirn (% lesional HIV)	Risk ratio(95% CI)	Adjusted risk ratio^1^ (95% CI)
Total		N = 334	N = 333		
**All patients**	**667**	**89 (27%)**	**63 (19%)**	**0.71 (0.53–0.94)**	**0.73 (0.55–0.98)**
**Country**				P-int = 0.50	P-int = 0.26
Ghana Females	70	1 (3%)	1 (3%)	-2	-2
CAR Females	94	21 (39%)	15 (38%)	0.96 (0.57–1.63)	0.87 (0.48–1.59)
Malawi Females	58	2 (8%)	2 (6%)	-2	-2
Malawi Males	135	16 (26%)	17 (23%)	0.90 (0.50–1.64)	1.00 (0.54–1.83)
South Africa Males	310	59 (31%)	28 (18%)	0.58 (0.38–0.87)	0.58 (0.39–0.84)
**By ulcer aetiology**				P-int = 0.33^3^	P-int = 0.22^3^
Non HSV ulcer	200	20 (21%)	20 (19%)	0.90 (0.51–1.56)	0.96 (0.52–1.76)
HSV ulcer	466	69 (29%)	43 (19%)	0.66 (0.47–0.92)	0.66 (0.48–0.92)
HSV-2 ulcer	465	68 (29%)	43 (19%)	0.66 (0.47–0.93)	0.66 (0.48–0.92)
First episode HSV-2 ulcer	64	9 (27%)	2 (7%)	0.24 (0.05–1.02)	0.20 (0.03–1.11)
Recurrent HSV-2 ulcer	401	59 (29%)	41 (21%)	0.73 (0.51–1.03)	0.74 (0.52–1.02)
**By duration before presentation**				P-int = 0.56	P-int = 0.32
<2 days	33	3 (18%)	1 (6%)	0.35 (0.04–3.17)	0
3–4 days	167	16 (18%)	9 (11%)	0.61 (0.29–1.31)	0.57 (0.26–1.24)
5–6 days	121	19 (30%)	9 (16%)	0.51 (0.25–1.05)	0.44 (0.21–0.92)
≥7 days	337	51 (31%)	44 (25%)	0.82 (0.58–1.15)	0.88 (0.63–1.23)
**By size of ulcer at baseline**				P = 0.52	P-int = 0.33
<50 mm^2^	364	33 (19%)	22 (12%)	0.64 (0.39–1.05)	0.55 (0.32–0.93)
> = 50 mm^2^	303	56 (36%)	41 (28%)	0.78 (0.56–1.09)	0.79 (0.57–1.11)
**By CD4 status (cells/mm^3^)**				P-int = 0.06	P-int = 0.12
CD4≥500	92	5 (10%)	7 (16%)	1.60 (0.54–4.69)	1.38 (0.54–3.50)
CD4 200–500	257	34 (29%)	18 (13%)	0.46 (0.27–0.77)	0.48 (0.29–0.80)
CD4<200	226	37 (32%)	29 (27%)	0.84 (0.56–1.27)	0.87 (0.62–1.23)

1 Adjusted for all other variables in the table.

2 Insufficient data to estimate risk ratio and 95% CI.

3 Interaction between non-HSV and HSV ulcers.

Among the 466 HIV-1 seropositive individuals with HSV ulcers, there was strong evidence of an effect on the intervention on reducing prevalence of detectable lesional HIV-1 RNA (aRR = 0.66, 95% CI 0.47–0.92) and this was greater among the 64 participants with first episode HSV-2 ulcers (aRR = 0.20, 95% CI 0.03–1.11) compared to the effect among the 401 participants with recurrent HSV-2 ulcers (aRR = 0.74, 95% CI 0.52–1.02), although this difference was not significant (p-interaction = 0.13). The impact on both types of HSV-2 ulcers was stronger in South Africa, although the differences were not significant.

The impact on lesional HIV-1 RNA was also stronger in those presenting within a week of symptoms (RR = 0.54, 95% CI 0.33–0.90) and those with small ulcers (RR = 0.55, 95% CI 0.31–0.87). Overall, there was no evidence of an impact among the patients with CD4 counts above 500 cells/mm^3^ or below 200 cells/mm^3^, but strong evidence of reduced lesional HIV-1 RNA associated with acyclovir among those with CD4 counts between 200 and 500 cells/mm^3^ (adjusted RR = 0.48, 95% CI 0.29–0.80). There was no evidence of interaction by site, but in the South African patients, there was a protective effect of acyclovir in each CD4 group (CD4>500 cells/mm^3^: RR = 0.69, 95% CI 0.11–4.31; CD4 200–500 cells/mm^3^: RR = 0.34, 95% CI 0.17–0.68; CD4<200 cells/mm^3^: RR = 0.84, 95% CI 0.53–1.32). By contrast, little effect was observed in the non-South African patients (CD4>500 cells/mm^3^: RR = 3.47, 95% CI 0.33–36.36; CD4 200–500 cells/mm^3^: RR = 0.74, 95% CI 0.35–1.57; CD4<200 cells/mm^3^: RR = 0.87; 95% CI 0.48–1.55).

## Discussion

Herpes simplex virus type 2 is a recognised and important co-factor of the HIV epidemic [1], [12]–[14]. A number of RCTs have failed to demonstrate an impact of HSV suppressive treatment on acquisition [15]–[16] or transmission [17] of HIV, although acyclovir or valacyclovir have been shown to decrease plasma and genital HIV levels [18]–[24] and even to modestly reduce HIV disease progression [25]. A meta-analysis of 8 RCTs showed that high dose acyclovir (≥3200 mg/day) significantly reduced mortality among HIV-infected individuals [26]. In this pooled analysis of episodic therapy trials, there was a small impact of acyclovir on the proportion of ulcers healed on day 7 and this was more substantial among patients with HSV ulcers, first episode HSV-2 ulcers, small ulcers, and HIV-1 seropositive individuals with CD4 counts between 200 and 500 cells/mm^2^. However, the impact was consistently greater in South Africa than in other sites, and this was not explained by different distributions of these clinical characteristics.

It is challenging to explain the greater impact of acyclovir on ulcer healing and lesional HIV in South Africa compared with other sites. The acyclovir regimen was the same in South Africa as in Ghana and Central African Republic (400 mg three times daily for 5 days), and lower than in Malawi (800 mg twice daily for 5 days); the duration of GUD before treatment was marginally shorter in South Africa, but comparable to Ghana (median 5 days vs. 7 days elsewhere); and ulcer characteristics were not different in other respects (size and number) between South Africa and other sites. Reported adherence was high in all three trials (>90% of participants reporting having taken all their tablets by day 7), although no information was available on drug concentrations, nor on drug quality and storage. A limitation of the trials was that the first visit date at which all patients were recalled was one week after randomization, and we cannot evaluate impact on healing within the first week. A number of possible explanations have been put forward for the lack of efficacy of suppressive therapy on HIV acquisition and transmission, which include the inadequacy of current HSV treatment regimens to properly control HSV-2 replication itself [17]. It is possible that such a mechanism could affect ulcer healing as well. In addition, these trials have shown that African patients tend to come late for the management of their ulcers, which would compromise the efficacy of antiviral therapy. Clearly, earlier provision of more potent, and perhaps more prolonged, HSV-2 therapies are required to control HSV-2 replication and hasten healing, particularly among HIV-positive patients [27]. Earlier provision would be facilitated by counselling of patients with recurrent genital herpes to improve symptom recognition, as has been successful in the United States [28].

This pooled analysis provides descriptive data on a large series of genital ulcer patients from four countries in sub-Saharan Africa. Almost two-thirds of ulcers were due to HSV-2, and of these, a quarter (225/918), were first-episode HSV-2 ulcers, representing also 16% of all GUD. Only 10% of all ulcers had a bacterial aetiology, alone or combined with HSV, although this varied by country. HIV-1 prevalence was high in these populations (58%), and a large proportion had CD4 counts below 200 cells/mm^3^, while few individuals were on antiretroviral therapy at the time of enrolment in 2003–2006.

The three trials were pragmatic trials designed to evaluate the effectiveness of adding acyclovir to current syndromic management of GUD, rather than the efficacy of acyclovir on treating herpetic ulcers *per se*. Although it is clear that there was a benefit in South Africa, the overall results suggest that there may be limited value in adding acyclovir to GUD syndromic management in some other African settings. Approximately seventeen GUD patients would need to be treated with acyclovir for one additional patient to be healed on day 7. There is likely to be some benefit in settings where a high proportion of ulcers are genital herpes, especially first episode HSV-2 ulcers which responded best to treatment, but this clinical and lab-based information is rarely available in resource-poor settings. The stronger effect among HIV-1 infected individuals than HIV uninfected individuals overall also suggests that acyclovir may be beneficial for GUD patients known to be HIV-infected at the time of presentation to clinical services. The high prevalence in this population highlights that genital ulceration in patient with unknown HIV status provides a potential entry point for provider-initiated HIV testing and care.

It is not possible to diagnose HSV infection easily, quickly and cheaply, let alone primary HSV-2 ulcers, with current tools in resource-constrained clinical settings. Given the limited effectiveness of adding acyclovir to syndromic management overall, and the lack of clear reasons for why the regimen was more effective in one trial than the other two, further research is needed to explore better options for the management of GUD patients in sub-Saharan Africa.
